# Isolated Infraspinatous Atrophy from a Spinoglenoid Cyst: A Case Report

**DOI:** 10.5704/MOJ.2203.024

**Published:** 2022-03

**Authors:** DN Gomez, NF Zulkahini, AR Ahmad, GN Solayar

**Affiliations:** 1 Department of Orthopaedic Surgery, Hospital Tuanku Ja'afar, Seremban, Malaysia; 2 Department of Sports Medicine, Hospital Tuanku Ja'afar, Seremban, Malaysia; 3 Department of Orthopaedic Surgery, International Medical University, Seremban, Malaysia

**Keywords:** spinoglenoid cyst, infraspinatous atrophy, superior labral anterior to posterior (SLAP) tear

## Abstract

We present a case of a 26-year-old gentleman with isolated right infraspinatus atrophy arising from a spinoglenoid cyst of the right shoulder. He presented two years following his shoulder injury and failed conservative rehabilitation alone. At initial arthroscopic surgery, a superior labral anterior to posterior (SLAP) tear was diagnosed and the spinoglenoid cyst was debrided without formal labral repair. The patient’s condition did not improve, and second arthroscopy was performed three months following the first with suture anchor repair of the labral tear and cyst decompression. Post-operative magnetic resonant imaging (MRI) scans showed complete resolution of the cyst and recovery of infraspinatus muscle bulk at six months. At final follow-up 18 months post SLAP repair, he has regained full shoulder function and has returned to recreational sports. Our case highlights the importance of proper management of SLAP tears in resolving spinoglenoid cysts by demonstrating the outcomes from two different surgical methods in the same patient.

## Introduction

Paralabral ganglion cyst, frequently reported along the posterior, superior, and anterior aspects of the glenohumeral joint are a rare cause of shoulder pain. It rarely becomes evident clinically unless they cause compression of surrounding structures^1^.

We would like to present a case of a shoulder paralabral ganglion cyst causing suprascapular nerve compression at the spinoglenoid notch resulting in atrophy of the infraspinatus muscle. Outcomes following arthroscopic decompression alone versus arthroscopic labral repair are also discussed in this report.

## Case Report

A 26-year-old gentleman initially presented to our orthopaedic clinic with complaints of worsening right shoulder pain and weakness. Two years prior, he developed a sharp pain in his right shoulder after lifting a heavy object but did not seek medical treatment at that time. His pain initially subsided following two months of rest. Unfortunately, he experienced increasing weakness in the right shoulder associated with intermittent bouts of discomfort over the duration till presentation.

On initial right shoulder examination, there was atrophy of the infraspinatus fossa with evidence of scapular dyskinesia. There was global reduction in active range of motion compared to the contralateral shoulder. External rotation against resistance (infraspinatus) demonstrated marked weakness compared to the other rotator cuff muscles.

Plain radiographs of his right shoulder were unremarkable. He was initial treated with rehabilitation which involved stretching and strengthening of his rotator cuff, deltoid, and scapular muscles. Dry needling therapy and electrical stimulation was also performed to address the weak infraspinatus muscle. He was compliant to his rehabilitation measures for three months; however, progress was unremarkable.

**Fig. 1:**
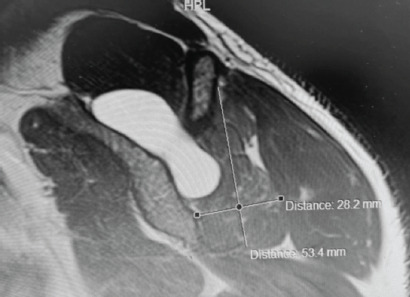
T2 weighted image showing a spinoglenoid cyst over the supraspinous fossa of the shoulder. Cruciform measurements show the presence of infraspinatus muscle atrophy.

Following initial rehabilitation, an MRI of his right shoulder was performed which showed a large spinoglenoid cyst located over the supraspinous fossa with marked atrophy of the infraspinatus and sparing of the supraspinatus muscle. (Fig. 1)

Bedside aspiration under ultrasound guidance was attempted; however, this was unsuccessful due to thick consistency of the cystic contents. He subsequently underwent shoulder arthroscopy which demonstrated a SLAP tear which was not reported in the initial MRI. The cyst was debrided intra-articularly through the labral defect and the superior glenoid rim was decorticated to promote labral healing. Formal repair of the SLAP lesion was not performed during this operation as suitable arthroscopic repair anchors were unavailable.

**Fig. 2:**
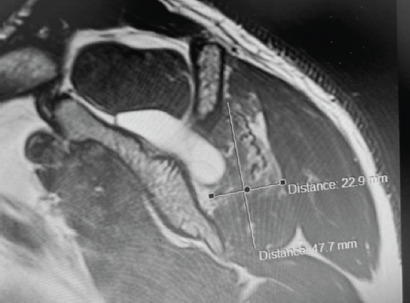
T2 weighted image showing a persistent spinoglenoid cyst of the shoulder following initial surgery. There is further atrophy of the infraspinatus muscle compared to the earlier MRI pre-operatively.

Post-operatively, there was mild improvement in pain however, his infraspinatus weakness remained. A subsequent Magnetic Resonance Arthrography (MRAs) three months following surgery confirmed the SLAP tear, re-accumulation of the spinoglenoid cyst and persistent infraspinatus atrophy (Fig 2). After appropriate patient counselling, a second arthroscopic surgery was performed with instruments specific for labral repair prepared in advance. The cyst was again debrided, and the SLAP lesion formally repaired with three anchor sutures.

**Fig. 3:**
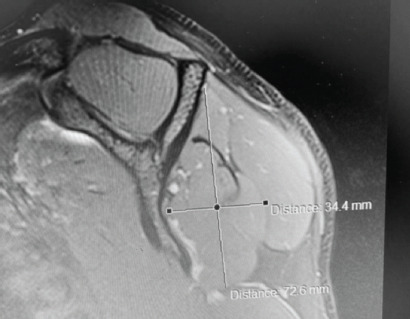
MRI showing complete resolution of the cyst and recovery of infraspinatus muscle bulk.

Post-operatively, the patient recovered well. His symptoms of pain resolved with significant improvement of external rotation strength. He regained full range of motion of his right shoulder and the muscle bulk over the infraspinatus fossa improved. A repeat MRI done six months following SLAP repair confirmed full resolution of the spinoglenoid cyst and restoration of infraspinatus muscle bulk (Fig. 3). At final follow-up 18 months post-surgery, the patient has made a full functional recovery and has returned to recreational sports.

## Discussion

Spinoglenoid cysts of the shoulder are frequently associated with labral tears with a reported incidence of up to 89%^1^. Problems with these cysts include pain and nerve compression resulting in isolated or combined degeneration of the supra and infraspinatus muscles.

Treatment of suprascapular nerve entrapment of the shoulder consists of conservative or surgical options. Most authors agree that in the absence of a space-occupying lesion, treatment should be conservative. In the presence of a space occupying lesion causing symptomatic nerve compression, the treatment involves surgical exploration and excision. Needle aspiration alone has an unsatisfactorily high recurrence rate of up to 48% in 2 years^2^.

Traditionally, open paralabral cysts excision has been shown to produce good results. Open procedures allow for direct visualisation of the cyst and the suprascapular nerve. However, this is associated with increased morbidity due to the larger incision and extensive muscle detachment. Conversely, arthroscopic decompression of paralabral cysts has shown to produce similar results to open surgery without the adverse outcomes associated with extensive surgery. Patients who underwent surgical decompression of the cyst with labral defect fixation report the highest satisfaction outcomes in most cases^3^.

There have been reports where spinoglenoid cyst debridement alone without the need for labral repair have been successful. In their cohort of patients by Kim *et al*, 57% of their patients had either a type 1 or a non-demonstratable tear at arthroscopy which negated the need for repair. We do note however that their cyst decompression was performed via a sub-acromial approach^4^. In our case, the intra-articular decompression performed (as opposed to a sub-acromial approach) may have worsened the SLAP lesion and contributed to why our first operation failed.

The initial MRI report which did not specifically report a SLAP tear may have contributed to our failure to prepare when faced with the lesion intra-operatively. Our experience highlights the importance of a high index of suspicion towards these tears and the appropriate ability to arthroscopically repair these lesions though the initial MRI reports may be negative. MRAs have been shown to have better sensitivities and higher accuracies detecting SLAP tears compared to MRIs alone^5^. In hindsight, an MRA may have identified the SLAP lesion initially and hence, improved our preparation for the SLAP repair at first surgery.

In conclusion, this case reinforces the importance of diagnosing and repairing a SLAP tear in the setting of a spinoglenoid cyst causing isolated infraspinatus atrophy. In our experience, intra-articular decompression alone without labral repair is insufficient and one might need to consider a sub-acromial approach to cyst decompression in this setting. Successful patient outcomes are achievable with proper spinoglenoid cyst decompression and SLAP repair with subsequent restoration of infraspinatus muscle bulk and function.
